# The transcriptomic and epigenetic alterations in type 2 diabetes mellitus patients of Chinese Tibetan and Han populations

**DOI:** 10.3389/fendo.2023.1122047

**Published:** 2023-02-16

**Authors:** Xian Wang, Jie Liu, Qiuhong Wang, Qiu Chen

**Affiliations:** ^1^ School of Biological and Behavioral Sciences, Queen Mary University of London, London, United Kingdom; ^2^ Department of Endocrinology, Hospital of Chengdu University of Traditional Chinese Medicine, Chengdu, China; ^3^ Department of Endocrinology, Kunming Municipal Hospital of Traditional Chinese Medicine, Kumning, China; ^4^ Department of Endocrinology, Guang’anmen Hospital, China Academy of Chinese Medical Sciences, Beijing, China

**Keywords:** DNA methylation, type 2 diabetes mellitus, Han, Tibetan, transcriptome

## Abstract

**Background:**

Due to the distinctive living environment, lifestyle, and diet, the Tibetan community in China has the lowest prevalence of T2DM and prediabetes among numerous ethnic groups, while Han community shows the highest statistic. In this study, we aim to conclude the clinical manifestations of both Tibetan and Han T2DM patients and their association with transcriptomic and epigenetic alterations.

**Methods:**

A cross-sectional study including 120 T2DM patients from Han and Tibetan ethnic groups were conducted between 2019 to 2021 at the Hospital of Chengdu University of Traditional Chinese Medicine. The various clinical features and laboratory tests were recorded and analyzed between the two groups. The genome-wide methylation pattern and RNA expression were determined by Reduced Representation Bisulfite Sequencing (RBBS) and Poly (A) RNA sequencing (RNA-seq) from leucocytes of peripheral blood samples in 6 Han and 6 Tibetan patients. GO analysis and KEGG analysis were conducted in differentially expressed genes and those with differentially methylated regions.

**Results:**

Compared to Han, Tibetan T2DM individuals intake more coarse grains, meat and yak butter, but less refined grains, vegetables and fruit. They also showed increased BMI, Hb, HbA1c, LDL, ALT, GGT and eGFR, and decreased level of BUN. Among the 12 patients in the exploratory cohort, we identified 5178 hypomethylated and 4787 hypermethylated regions involving 1613 genes in the Tibetan group. RNA-seq showed a total of 947 differentially expressed genes (DEGs) between the two groups, with 523 up-regulated and 424 down-regulated in Tibetan patients. By integrating DNA methylation and RNA expression data, we identified 112 DEGs with differentially methylated regions (overlapping genes) and 14 DEGs with promoter-related DMRs. The functional enrichment analysis demonstrated that the overlapping genes were primarily involved in metabolic pathways, PI3K-Akt signaling pathway, MAPK signaling pathway, pathways in cancer and Rap1 signaling pathway.

**Conclusion:**

Our study demonstrates the clinical characteristics of T2DM differ subtly between various ethnic groups that may be related to epigenetic modifications, thus providing evidence and ideas for additional research on the genetic pattern of T2DM.

## 1 Introduction

The main features of type 2 diabetes mellitus (T2DM) include hyperinsulinemia, insulin resistance (IR) and islet cell damage, which can reach 50% at the time of diagnosis (1). With a high-energy diet, decreased physical activity, and an increase in obesity, the incidence of diabetes is rising globally, along with the rate of disability and mortality. People who have T2DM experience vascular and neurological consequences, as well as life, psychological, and financial stress. The diabetic population will predictably reach 147 million by 2045 (2). Most diabetes is a complex disease caused by a combination of multiple genes and environmental factors. Genetic factors are present in approximately 25% to 69% of people with T2DM worldwide (3) and over 560 genetic loci are identified to be relevant (4).

Epigenetics, including DNA methylation, histone modifications and microRNAs, lead to changes in gene function based on mitosis and meiosis without alteration in DNA sequence (5), in which DNA methylation has been recognized to be an important genetic factor contributing to T2DM (6). DNA methylation refers to the S-adenosyl methionine (SAM), as the methyl donor, transfers the activated methyl group to carbon 5 of the cytosine-phosphate-guanine (CpG) by the catalyzation of DNA methyltransferases (DNMTs). In general, gene expression is opposite to the level of methylation in the promoter region, which means low methylation levels result in up-regulation of gene expression, whereas high methylation results in down-regulation of expression (7, 8). As DNA methylation is reversible and can be interfered with, some chemicals can be used as targets to modify DNA methylation (9), providing a new perspective for T2DM treatment. Previous studies have shown that many genes are related to islet function, such as PDX1 (10), PPARGC1A (11), INS (12), GLP1R (13) and KCNQ1 (14), have been associated with the development of T2DM. Meanwhile, methylome-wide association studies (MWAS) for T2DM have identified differentially methylated sites (DMSs) in TXNIP (15), PHOSPHO1 (16), SREBF1 (17), ABCG1 (17), SOCS3 (18), and CPTA1 (19).

Environmental factors such as diet, exercise and obesity can also alter the epigenome. Tibetans are a distinct ethnic group in China that have historically lived at high altitudes. They primarily reside in the Tibetan Autonomous Region (TAR), as well as the provinces of Qinghai, Sichuan, Yunnan, and Gansu in China. Although highlanders had a lower incidence of diabetes, the number has quickly risen as a result of greater longevity and lifestyle changes (20, 21). According to nationwide research, the Han Chinese population had a 14.7% prevalence of diabetes and a 38.8% prevalence of prediabetes, whereas the Tibetan community had the lowest prevalence of both conditions at 4.3% and 31.3%, respectively (22). Lifestyle changes, particularly in calorie intake, are associated with the development of diabetes, possibly through epigenetic mechanisms (23, 24). This study aimed to demonstrate the differences in clinical characteristics between Tibetan and Han T2DM patients and to explore the transcriptomic and epigenetic alterations in the two groups.

## 2 Materials and methods

### 2.1 Study population

#### 2.1.1 Cross-sectional cohort

A total of 60 Tibetan and 60 Han patients with T2DM were recruited at the Hospital of Chengdu University of Traditional Chinese Medicine from 2019 to 2021. All the patients were diagnosed with T2DM according to the 1999 WHO criteria (25). There was no kinship between the included study subjects and three consecutive generations for each patient are the same ethnic group. The exclusion criteria include 1) other types of diabetes; 2) having immune system diseases; 3) any types of tumors; 4) acute and chronic infections; 5) psychoneurological disorders; 6) recent use of drugs that affect lipid metabolism; 7) having liver or kidney failure or severe heart diseases; 8) disagreeing to participate in the study.

#### 2.1.2 Subjects enrolled for exploratory cohort

Among the cross-sectional cohort, 6 Tibetan and 6 matched Han T2DM patients were selected for the exploratory cohort of RNA expression and DNA methylation. These two groups were selected by a matched pairs design based on shared characteristics including age, gender, weight, height and duration of T2DM to control lurking variables.

### 2.2 Clinical data collection

The general information for all patients included age, gender, body mass index (BMI), drinking and smoking history, family history of diabetes, the duration of T2DM, food intake, systolic blood pressure (SBP), diastolic blood pressure (DBP) and hemoglobin (Hb). In addition to HbA1c test, a standard 2-h OGTT test was performed by using a 75g glucose load to assess the patient’s islet function and glycemic control. Plasma glucose and insulin level at 0 (fasting), 1, 2 and 3-hour postprandial blood glucose (PBG) were measured, and blood C-peptide was measured at 0 and 2-hour postprandial. The biochemical analysis includes the total cholesterol (TC), triglyceride (TG), high-density lipoprotein cholesterol (HDL-C), low-density lipoprotein cholesterol (LDL-C), alanine aminotransferase (ALT), aspartate aminotransferase (AST), alkaline phosphatase (ALP), γ-glutamyltransferase (GGT), total bile acid (TBA), direct bilirubin (DBIL), blood creatinine (BCr), blood urea nitrogen (BUN), blood uric acid (BUA), were detected to estimate blood lipids, liver function, and kidney function. The Cockcroft-Gault equation was used to determine the estimated glomerular filtration rate (eGFR) (26).

### 2.3 Blood sample and DNA extraction

In the exploratory cohort, 3ml EDTA-treated peripheral blood sample of each participant was collected and stored in -80°C. Genomic DNA was extracted from peripheral blood using magnetic universal genomic DNA kit (TIANGEN Biotech (Beijing) co., Ltd). DNA concentration and quality were measured by Nanodrop.

### 2.4 Reduced representation bisulfite sequencing

1µg genomic DNA was digested using MspI enzyme for 16 hours at 37°C. After digestion, libraries were constructed as the Illumina Pair-End protocol with some modifications. Briefly, purified digested DNA was subsequently treated with a mix of T4 DNA polymerase, Klenow Fragment and T4 polynucleotide kinase to repair, blunt and phosphorylate ends. The DNA fragments were subsequently 3’ adenylated using Klenow Fragment (3’-5’ exo-) and following with ligation to adaptors synthesized with 5’-methylcytosine instead of cytosine using T4 DNA Ligase. the DNA was purified using QIAquick PCR purification kit (Qiagen) after reaction of each step. After purification, the library was subjected to 40°C for 30 min treatment in a thermo cycler with the lid heated at 57°C. After that, centrifuged the reaction mixture at 14,000 X g for 10 min and then transferred the supernatant into a new 0.2 ml PCR tube for the further bisulfite treatment, respectively. Bisulfite conversion treatment was performed using a ZYMO EZ DNA Methylation-Gold Kit (Zymo research, Irvine, CA, USA) according to the manufacturer’s instructions. The final RRBS libraries were generated by PCR amplification using adapter compatible barcode primers, quantified by an Agilent 2100 Bioanalyzer (Agilent Technologies) and real-time PCR assay and then sequenced by Illumina Hiseq.

### 2.5 Methylation calculation and identification of DMRs

Low-quality reads that contained more than 5 ‘N’s or had a low-quality value for over 50% of the sequence (Phred score< 5) were filtered. The sequencing reads of the samples were aligned to the human reference genome (hg19) using BSMAP (Version 2.74) (27). The methylated CpG (mCG) sites were identified following a previously described algorithm (28). The methylation levels for each sample were calculated using in-house Perl scripts. Differentially methylated regions (DMRs) were identified using metilene (Version 0.2-6) within a 500 bp sliding window at 250 bp steps with at least 10 CpGs covered by over 10× sequence reads, applying the thresholds of differential methylation β ≥ 15%, FDR for two-dimensional Kolmogorov-Smirnov-Test p-value< 0.05 (29). The enrichment analyses were conducted using WebGestalt (WEB-based Gene SeT Analysis Toolkit) (30).

### 2.6 RNA library construction and sequencing

Total RNA was extracted from cells using Trizol (Invitrogen) according to the manufacturer’s protocol, and ribosomal RNA was removed using the Ribo-Zero™ kit (Epicentre, Madison, WI, USA). Fragmented RNA (the average length was approximately 200 bp) was subjected to first-strand and second-strand cDNA synthesis followed by adaptor ligation and enrichment with a low cycle according to instructions of NEBNext^®^ Ultra™ RNA Library Prep Kit for Illumina (NEB, USA). The purified library products were evaluated using the Agilent 2200 TapeStation and Qubit^®^2.0 (Life Technologies, USA). The libraries were paired-end sequenced (PE150, Sequencing reads were 150 bp) at Guangzhou MethylGene Co., Ltd. (Guangzhou, China) using the Illumina Xten platform.

### 2.7 Pre-processing of sequencing reads/quality control

Raw fastq sequences were treated with Trimmomatic tools (v 0.36) using the following options: TRAILING: 20, MINLEN:235 and CROP:235, to remove trailing sequences below a Phred quality score of 20 and to achieve uniform sequence lengths for downstream clustering processes. Sequencing read quality was inspected using the FastQC software. Adapter removal and read trimming were performed using Trimmomatic. Sequencing reads were trimmed from the end (base quality less than Q20) and filtered by length (less than 25).

### 2.8 Quantification of gene expression level

Paired-end reads were aligned to the human reference genome (hg19) with HISAT2. HTSeq v0. 6.0 was used to count the numbers of reads mapped to each gene. The whole sample expression levels were presented as RPKM (expected number of Reads Per Kilobase of transcript sequence per Million base pairs sequenced), which is the recommended and most common method to estimate the level of gene expression.

### 2.9 Differential expression analysis

The statistically significant DE genes were obtained by an adjusted P-value threshold of<0.05 and |log2(fold change) | > 1 using the DEGseq software. Finally, a hierarchical clustering analysis was performed using the R language package gplots according to the RPKM values of differential genes in different groups. And colors represent different clustering information, such as the similar expression pattern in the same group, including similar functions or participating in the same biological process.

### 2.10 GO terms and KEGG pathway enrichment analysis

All differentially expressed mRNAs were selected for GO and KEGG pathway analyses. GO was performed with KOBAS3.0 software, including cellular component (CC), molecular function (MF) and biological process (BP). GO provides label classification of gene function and gene product attributes (http://www.geneontology.org). GO analysis covers three domains: cellular component (CC), molecular function (MF) and biological process (BP). The differentially expressed mRNAs and the enrichment of different pathways were mapped using the KEGG pathways with KOBAS3.0 software (http://www.genome.jp/kegg).

### 2.11 Statistical analysis

The median and quartile were used for the statistical and data description of the normally distributed measures, and the number of cases (n) and percentages (%) were used for the statistical and data description of the categorical counts. Normality and homogeneity of all data were evaluated by Kolmogorov-Smirnov test. Student T-test or Mann-Whitney U test was applied to compare the differences of continuous variables. Pearson Chi-square test was employed to evaluate statistical differences of categorical variables. The Wilcoxon test was used to compare the continuous non-normally distributed variables between 6 Tibetans and 6 Hans in the exploratory cohort. Pearson correlation was used to identify the 14 overlapping genes and clinical characteristics with significant differences. All data were statistically analyzed by SPSS 23.0 software (SPSS Inc., Chicago, IL, USA). Graphs were generated using Graphpad 7.0 software (GraphPad Software, Inc., San Diego, USA).

## 3 Results

### 3.1 The demographical and clinical characteristics between Tibetan and Han T2DM populations

**Figure 1:**
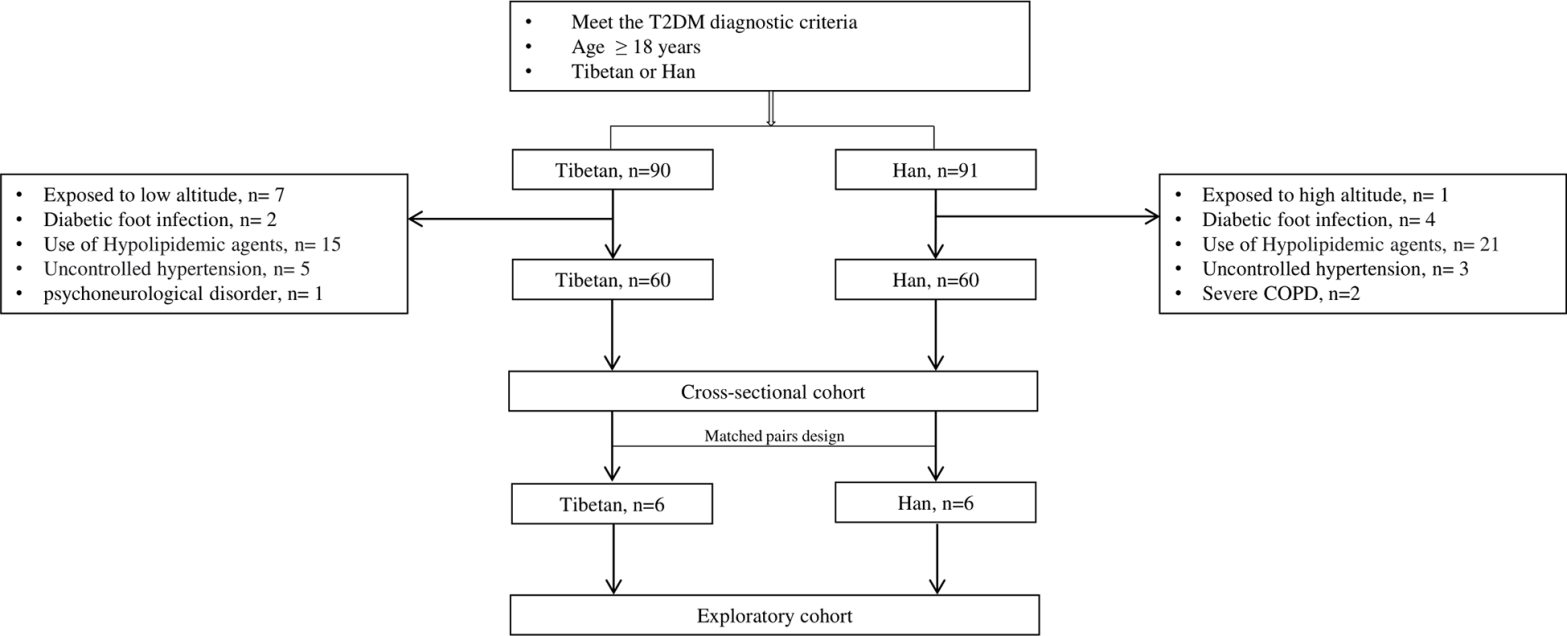
Flow chart of patient collection. COPD: chronic obstructive pulmonary disease.

A total of 120 participants were enrolled for the final analysis, including 60 Tibetans and 60 Hans. The patient flow chart is demonstrated in **Figure 1**. The basic and biochemical characteristics are shown in **Table 1**. Although no difference was observed in age, gender, and duration of T2DM, the BMI of Tibetans was significantly higher than Hans (26.08 vs 23.3, P = 0.017). Tibetans consume fewer refined grains (141.5 g/day vs 193.5 g/day, P< 0.001), vegetables and fruit (91 g/day vs 296.5 g/day, P< 0.001) than Han people, but they consume more coarse grains (171 g/day vs 63.5 g/day, P< 0.001), meat (181.5 g/day vs 100.5 g/day, P< 0.001), and yak butter (98.5 g/day vs 0 g/day, P< 0.001). Not surprisingly, the Hb level of Tibetans is higher than Hans (146.5 g/L vs 138.5 g/L, P< 0.001) due to the high-altitude, low-oxygen environment of Tibetan settlements. Despite similar BG, insulin, and C-peptide level, HbA1c of Tibetan T2DM patients was higher than Han patients (9.75% vs 8.65%, P = 0.001). Similarly, LDL level is significantly higher in Tibetan group compared to Han group (3.12 mmol/L vs 2.53 mmol/L, P = 0.002). Regarding the liver function, the blood tests also showed higher levels of ALT (30.5 IU/L vs 21.5 IU/L, P = 0.013), and GGT (38 IU/L vs 21 IU/L, P< 0.001). The level of BUN was lower (4.95 mmol/L vs 5.61 mmol/L, P = 0.002) and eGFR of Tibetan T2DM patients was statistically higher than Han patients (129.77 mL/min vs 96.5 mL/min, P< 0.001). There were no significant differences in other parameters of biochemical tests between the two groups.

**Table 1:** Demographical and biochemical characteristics between Tibetan and Han T2DM patients in cross-sectional cohort.

	Tibetan (n=60)	Han (n=60)	P value
Age (years)	49 (42.25-60)	53 (45-63)	0.163
Male (female)	34 (56.7%)	31 (51.7%)	0.583
BMI (kg/m^2^)	26.08 (23.63-28)	23.3 (21.88-25.19)	0.017*
Tabaco (n)	26 (43.3%)	22 (36.7%)	0.456
Alcohol (n)	31 (51.7%)	29 (48.3%)	0.715
Diabetes family history (n)	19 (31.7%)	22 (36.7%)	0.564
Duration of T2DM (years)	7 (3-11)	7 (3-12)	0.737
Food intake (g/day)			
Refined grains	141.5 (91.25-182.3)	193.5 (138.5-224.8)	<0.001*
Coarse grains	171 (46.5-80.75)	63.5 (46.5-80.75)	<0.001*
Meat	181.5 (142.3-219.8)	100.5 (74-121.8)	<0.001*
Vegetables and fruit	91 (71.25-110.8)	296.5 (231.3-377.5)	<0.001*
Yak butter	98.5 (65.75-126.8)	0 (0-7.25)	<0.001*
SBP (mmHg)	121.5 (110-133.25)	125 (117-143)	0.113
DBP (mmHg)	78 (70.25-85)	77.5 (70-85)	0.562
Hb (g/L)	146.5 (138.25-158)	138.5 (118.5-148.75)	<0.001*
HbA1c (%)	9.75 (8.23-11.8)	8.65 (7.13-10.63)	0.001*
FBG (mmol/L)	8.84 (7.37-8.84)	7.83 (5.99-9.42)	0.053
1-hr PBG (mmol/L)	15.26 (12.99-17.95)	15.86 (13.29-17.54)	0.836
2-hr PBG (mmol/L)	17.84 (15.55-20.75)	17.71 (14.97-20.98)	0.836
3-hr PBG (mmol/L)	16.99 (13.3-19.1)	16.58 (13.09-20.36)	0.774
0-hr Insulin (mIU/L)	7.64 (4.15-11.93)	7.06 (4.19-11.31)	0.661
1-hr Insulin (mIU/L)	21.46 (11.56-38.04)	25.35 (15.23-43.23)	0.183
2-hr Insulin (mIU/L)	25.08 (13.41-42.01)	29.71 (16.57-50.18)	0.062
3-hr Insulin (mIU/L)	18.99 (11.53-34.71)	23.89 (15.25-50.18)	0.317
0-hr C-peptide (nmol/L)	0.77 (0.59-1.05)	0.66 (0.51-0.97)	0.863
2-hr C-peptide (nmol/L)	1.4 (1.14-2.1)	1.71 (1.16-2.59)	0.171
TC (mmol/L)	4.55 (3.98-5.17)	3.96 (3.49-5.16)	0.170
TG (mmol/L)	1.28 (0.99-2.18)	1.45 (1.05-2.13)	0.601
HDL (mmol/L)	0.93 (0.85-1.09)	1.05 (0.85-1.34)	0.051
LDL (mmol/L)	3.12 (2.6-3.7)	2.53 (1.9-3.18)	0.002*
ALT (IU/L)	30.5 (17.75-39)	21.5 (16-28.75)	0.013*
AST (IU/L)	19.5 (15-25.25)	20.5 (17-23)	0.812
ALP (IU/L)	79 (64-104.25)	82 (64.25-100)	0.636
GGT (IU/L)	38 (23.75-59.5)	21 (16-30)	<0.001*
TBA (μmol/L)	3.2 (2.25-6.75)	4.55 (2.93-7.83)	0.051
DBIL (μmol/L)	12.65 (9.85-17.8)	11.95 (9.2-16.5)	0.486
BCr (μmol/L)	59.9 (55-66.8)	59.55 (49-69.78)	0.706
BUN (mmol/L)	4.95 (3.76-6.04)	5.61 (4.85-7.28)	0.002*
BUA (μmol/L)	299 (258-378)	308.5 (251.75-390)	0.894
eGFR (mL/min)	129.77 (97.81-155.2)	96.5 (77.87-117.1)	<0.001*

BMI, body mass index; SBP, Systolic blood pressure; DBP, diastolic blood pressure; Hb, hemoglobin; HbA1c, hemoglobin A1c; FBG, fasting blood glucose; PG, post-prandial blood glucose; TC, total cholesterol; TG, triglycerides; HDL, high-density lipoprotein; LDL, low-density lipoprotein; ALT, alanine aminotransferase; AST, aspartate transaminase; ALP, alkaline phosphatase; GGT, γ-glutamyl transpeptidase; TBA, total bile acid; DBIL, direct bilirubin; BCr, blood creatinine; BUN, blood urea nitrogen; BUA, blood uric acid; eGFR, estimated glomerular filtration rate. * P<0.05

A total of 12 patients with 6 in each group were selected by paired design for the exploratory cohort. The age ranged from 33 to 54 years old and the duration of T2DM ranged from 2 to 7.1 years. As shown in **Supplementary Table S1**, there were no significant differences in basic and biochemical parameters except HbA1c (9.9% vs 9%, P = 0.046), FBG (8.48 mmol/L vs 9.99 mmol/L, P = 0.028), 3-hr Insulin (12.11 mIU/L vs 21.07 mIU/L, P = 0.046), HDL (0.94 mmol/L vs 1.17 mmol/L, P = 0.046) and eGFR (143.7 mL/min vs 97 mL/min, P = 0.028).

### 3.2 Differentially methylated positions and regions

**Figure 2:**
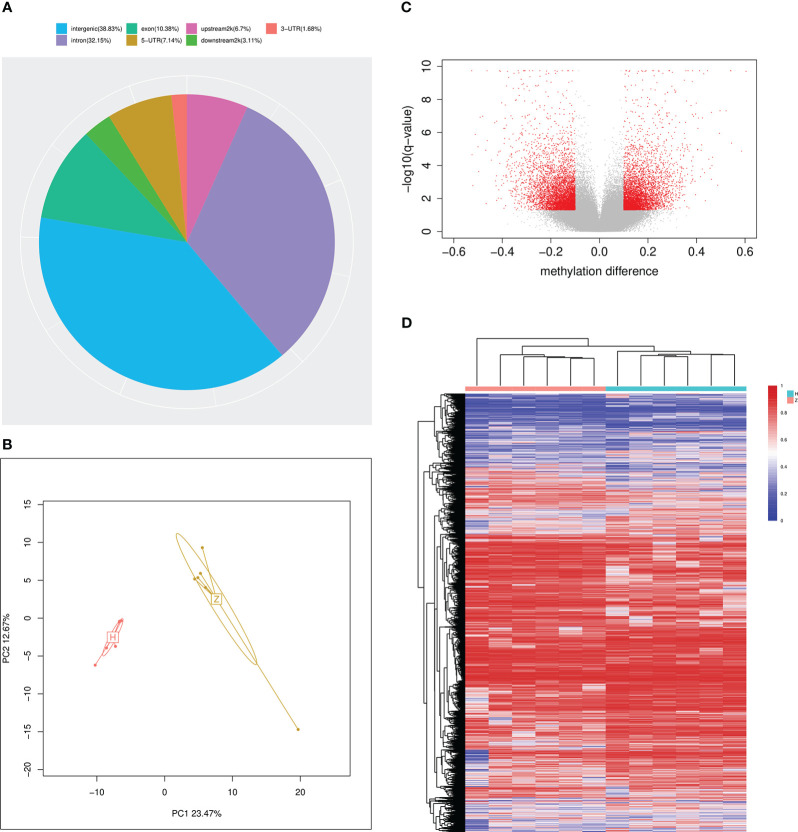
Summary of DMRs between Han and Tibetan T2D patients **(A)** The overall distribution of DMRs. **(B)** The principal component analysis plot using the differential methylated CpG sites between Han and Tibetans. **(C)** Volcano plot of methylation difference between Han and Tibetans. A total of 4787 CpG sites hypermethylated in Tibetans was represented by red point in the right side. A total of 5178 CpG sites hypomethylated in Tibetans was represented by red point in the left side. **(D)** Heatmap clustering analysis of DMRs of different gene functional regions. Highly methylated sites are shown in red and sparsely methylated sites are shown in blue. In addition, the pink clusters represent Tibetans and the blue clusters represent Han Chinese. H: Han, Z: Tibetan.

The whole-genome DNA methylation was detected by RRBS using peripheral blood samples from 6 Tibetan and 6 Han T2DM patients. After sulphite treatment, the conversion efficiency of all samples ranged from 98.82% to 99.27%. About 80% to 90% mCs were CG dinucleotides while about 10% to 20% were at CHG and CHH sites (G = A, C or T) (**Supplementary Figure S1**). Additionally, the methylation level of mC was around 80% to 100% while mCHG and mCHH were around 0% to 20%, with 20% as an interval (**Supplementary Figure S2**). We also explored the methylation levels in different genome regions. The level of methylation decreased in the 2kb upstream of transcription initiation but rose sharply in the exon region and reaches a maximum in the intron and 2kb downstream of genes (**Supplementary Figure S3**). The DMRs were mainly located in the intergenic region, accounting for 38.83%, followed by intron (32.15%) and exon regions (10.38%), respectively, in addition to 6.7% of DMRs within the gene promoter region (upstream 2kb) (**Figure 2A**). PCA found distinct clusters for study subjects (**Figure 2B**). The heatmap (**Figure 2C**) and volcano map (**Figure 2D**) have demonstrated the methylation difference between the two groups. Compared with Han group, we identified 5178 hypomethylated regions and 4787 hypermethylated regions in Tibetans (**Table 2**). 

**Table 2:** The numbers and length of differentially methylated regions.

Type	Number of DMRs	Number of cytosine	Length of DMR region
HypoDMR	5178	49,492	1,087,603
HyperDMR	4787	45,966	1,000,093

DMRs, differentially methylated regions; Hypo, hypomethylated; Hyper, hypermethylated.

**Figure 3:**
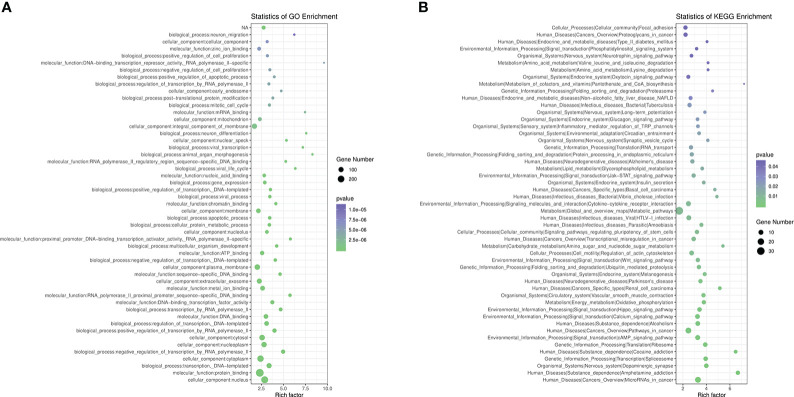
Pathway Analysis on DMR-related genes. **(A)**, GO analysis of DMR-related genes. **(B)**, KEGG analysis of DMR-related genes.

We performed GO functional analysis according to DMR-related genes, which were mostly enriched in protein binding (BP), nucleus (CC), cytoplasm (CC) and membrane (CC) (**Figure 3A**). KEGG analysis showed that DMR-related genes are mainly involved in metabolic pathway, pathways in cancer, cAMP signaling pathway, HTLV-I infection, cytokine-cytokine receptor interaction, calcium signaling pathway, alcoholism, regulation of actin cytoskeleton, hippo signaling pathway, Wnt signaling pathway, non-alcoholic fatty liver disease (NAFLD), insulin secretion, glycerophospholipid metabolism and type 2 diabetes mellitus (**Figure 3B**).

### 3.3 Transcriptome analysis

We conducted RNA-seq on peripheral blood samples from Han and Tibetan T2DM patients in order to investigate the relationship between DNA methylation and gene expression. Each sample produced about 8 giga bases (Gb) of filtered data. Additionally, using HISAT2 software, sequencing data were compared to the human reference genome with an average match rate of 90.2% per sample and an average unique mapping rate of 86.95% (**Supplementary Table S2**). Gene expression levels are calculated by RPKM as the number of reads per kilobase length from a given gene per million reads, and are calculated as $\;RPKM=\frac{Total\;exon\;reads}{Mapped\;reads\times exon\;length}\;$ (31).

**Figure 4:**
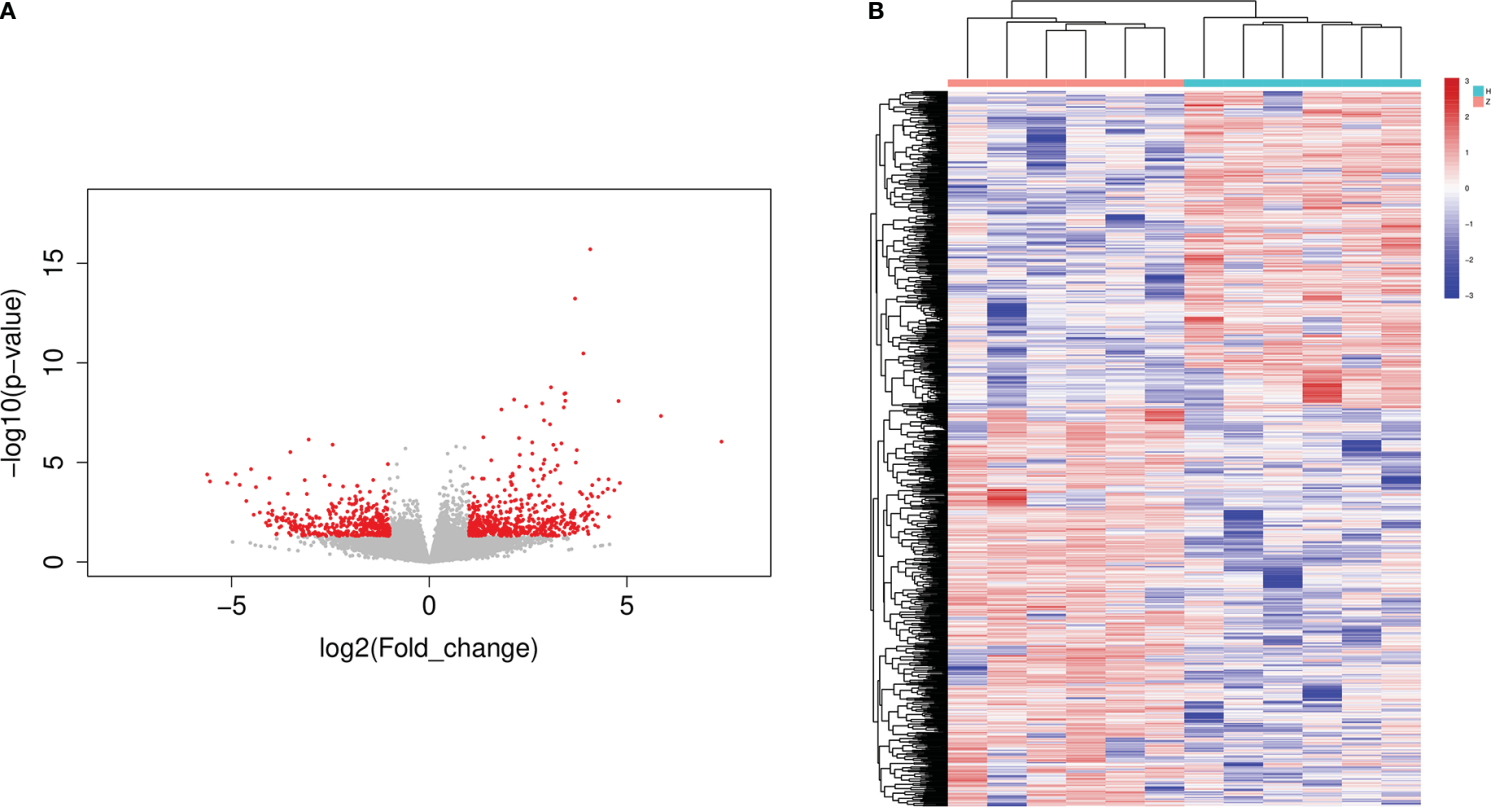
The volcano plot and heatmap of DEGs in Han and Tibetan T2D patients. **(A)** Volcano plot of DEGs. The x-axis represents the log2 fold change and the y-axis represents the log10 (P-value). The green dots represent downregulated genes and red dots represent upregulated genes. **(B)** Heat map of DEGs following clustering analysis. The vertical axis represents the sample, and the horizontal axis represents DEGs. Up: the number of up-regulated genes, down: the number of down-regulated genes, H: Han, Z: Tibetan.

A volcano map of significantly differentially expressed genes (DEGs) was created by differential gene expression analysis using the DESeq program, with 523 genes significantly up-regulated and 424 genes significantly down-regulated in the Tibetan group compared to the Han group (**Figure 4A**). The heat map revealed distinct gene expression patterns in the Tibetan and Han populations (**Figure 4B**).

**Figure 5:**
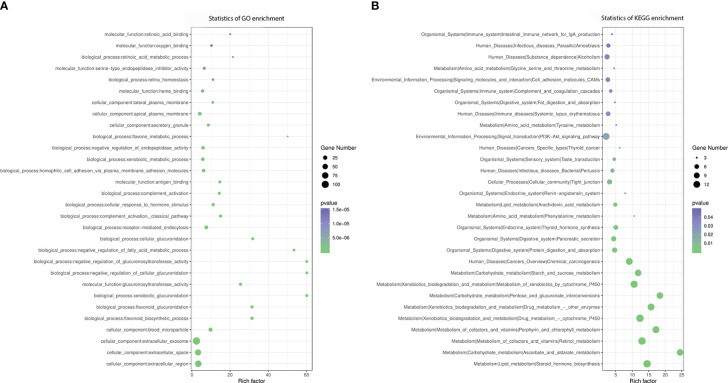
GO and KEGG enrichment analysis of DEGs. **(A)** GO analysis of differentially expressed genes, **(B)** KEGG analysis of differentially expressed genes.

Functional annotation showed that the most represented GO categories for DEGs were extracellular (CC), receptor-mediated endocytosis (BP), xenobiotic metabolic process (BP), negative regulation of endopeptidase activity (BP) and cellular response to hormone stimulus (BP) (**Figure 5A**), while KEGG enrichment analysis showed that the upregulated DEGs were mainly involved in steroid hormone biosynthesis, retinol metabolism, drug metabolism-cytochrome P450, PI3K-AkT signaling pathway, pentose and glucuronate interconversions, starch and sucrose metabolism, ascorbate and aldarate metabolism (**Figure 5B**).

### 3.4 Integrative analysis of transcriptome and DNA methylation

**Figure 6:**
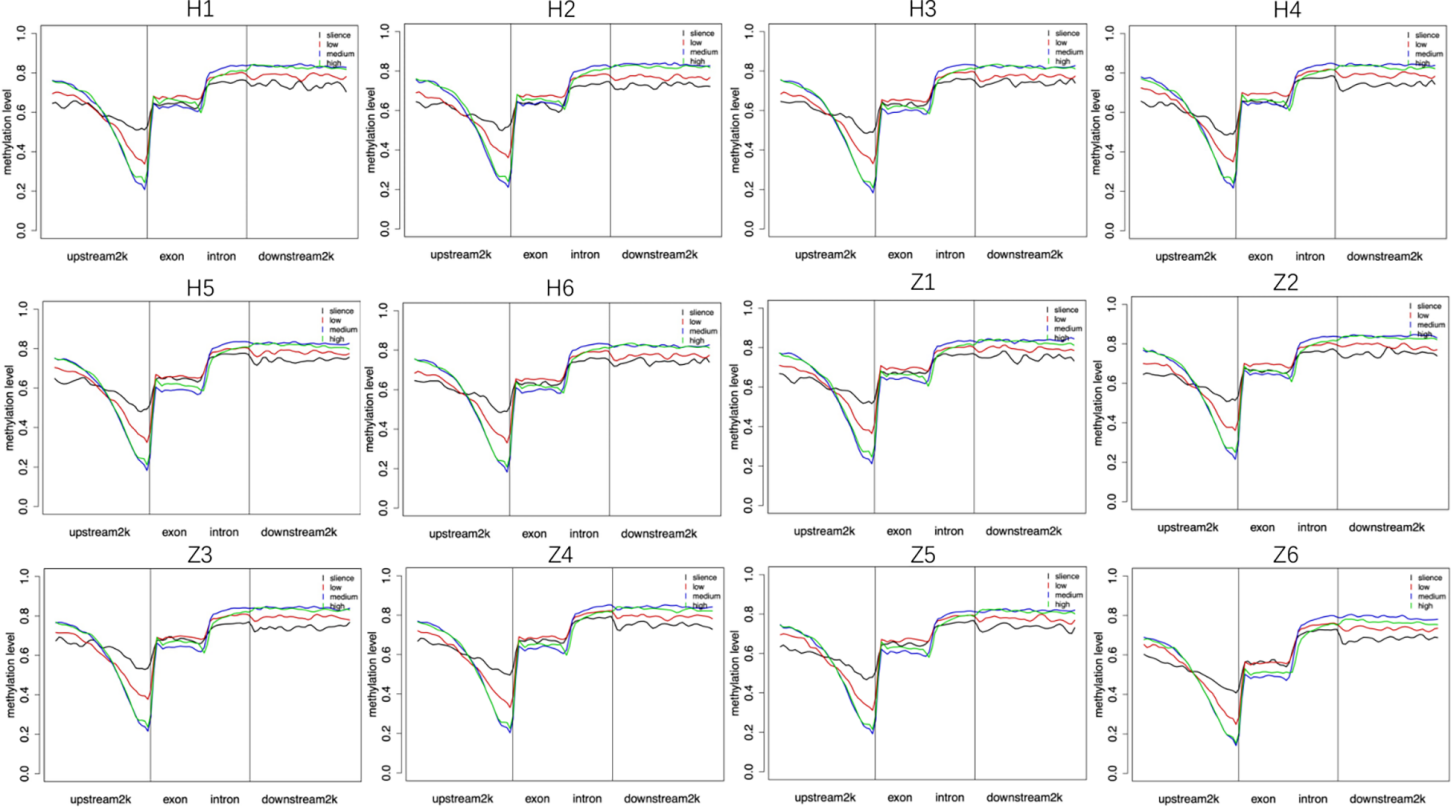
Relationship between DNA methylation and gene expression. Silence (RPKM=0), low: low expression (0<RPKM ≤ 1), medium: medium expression (1<RPKM ≤ 10), high: high expression (RPKM>10). H: Han, Z: Tibetan.

In general, gene expression is negatively correlated with DNA methylation. We divided each sample into four categories, including silence, low expression, medium expression, and high expression, according to the amount of gene expression and counted the methylation levels in the gene regions of each of the four categories of genes in a single sample. Our results showed DNA methylation was negatively correlated with gene expression in regions within 1k upstream of the gene, and genes with high methylation status were not expressed or were under-expressed (**Figure 6**).

**Figure 7:**
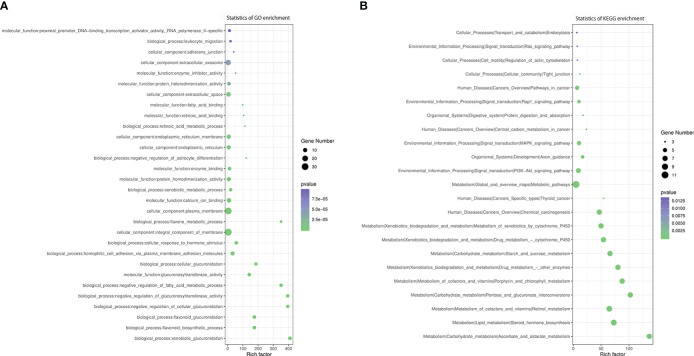
GO and KEGG enrichment analysis of DMR-related DEGs. **(A)** GO analysis of DMR-related DEGs. **(B)** KEGG analysis of DMR-related DEGs.

We observed 112 overlapping DEGs and DMR genes, of which 14 were promoter-related genes (**Table 3**). The GO enrichment analysis showed that the most significant enriched GO terms of overlapped genes are integral component of membrane (CC), plasma membrane (CC), homophilic cell adhesion *via* plasma membrane adhesion molecules (BP), calcium ion binding (BP) (**Figure 7A**).

**Table 3:** Overlapped genes of DEGs and promoter related DMR genes.

Gene	Location	log_2_ fold change	P value	Description	Gene type	Methylation H-Z
AJAP1	Chr1	1.689	0.016	adherens junctions associated protein 1	Protein coding	0.206577
APOB	Chr2	2.284	0.018	apolipoprotein B	Protein coding	0.1933
COL1A1	Chr17	1.516	0.025	collagen type I alpha 1 chain	Protein coding	0.35821
FOXA1	Chr14	3.105	0.006	forkhead box A1	Protein coding	0.119007
MIXL1	Chr1	-4.042	0.000	Mix paired-like homeobox	Protein coding	-0.17532
MYCN	Chr2	1.648	0.021	MYCN proto-oncogene, bHLH transcription factor	Protein coding	0.10136
OXCT2	Chr1	-1.395	0.040	3-oxoacid CoA-transferase 2	Protein coding	-0.19075
RHOD	Chr11	1.386	0.030	ras homolog family member D	Protein coding	0.14237
LAMA5-AS1	Chr20	-2.823	0.039	LAMA5 antisense RNA 1	LncRNA	-0.13274
LOC100134868	Chr20	-2.221	0.001	uncharacterized LOC100134868	LncRNA	-0.19718
LOC102723672	Chr7	-1.591	0.015	uncharacterized LOC102723672	LncRNA	-0.19339
LOC102723828	Chr4	-3.703	0.006	None	LncRNA	-0.11715
PAX8-AS1	Chr2	-1.557	0.045	PAX8 antisense RNA 1	LncRNA	-0.12671
UMODL1-AS1	Chr21	2.228	0.033	UMODL1 antisense RNA 1	LncRNA	0.11357

Methylation H-Z: the methylation level of Han minus that of Tibetan T2DM patients. H: Han, Z: Tibetan.

According to KEGG enrichment analysis, these overlapping genes were primarily involved in metabolic pathway, including metabolism of xenobiotics by cytochrome P450, steroid hormone biosynthesis, retinol metabolism, ascorbate and aldarate metabolism, pentose and glucuronate interconversions, porphyrin and chlorophyll metabolism, drug metabolism, starch and sucrose metabolism, chemical carcinogenesis, PI3K-Akt signaling pathway, MAPK signaling pathway, pathways in cancer and Rap1 signaling pathway (**Figure 7B**). The relationship between overlapping genes and significant clinical characteristics was analyzed by Pearson correlation analysis. We found that the HbA1c was associated with the expression of *RHOD* (R = 0.697, P< 0.05), *LOC100134868* (R = -0.697, P< 0.01) and *LOC102723828* (R = -0.661, P< 0.05); FBG was negatively associated with *APOB* (R = -0.631, P< 0.05); HDL was positively associated with *PAX8-AS1* (R = 0.615, P< 0.05); and eGFR was related with *FOXA* (R = 0.794, P< 0.01) and *UMODL1-AS1* (R = 0.662, P< 0.05). In addition, insulin levels at three hours after 75g glucose load test showed positive association with the expression of *MIXL1*, *OXCT2*, *LAMA5-AS1*, *LOC100134868* and *LOC102723672* while negatively related to *AJAP1*, as shown in **Supplementary table S3**.

## 4 Discussion

Our study reported the clinical characteristics of Han and Tibetan T2DM patients, indicating that the same disease has clinical differences between various ethnic groups and providing evidence for clinical individualization of T2DM treatment. We also revealed for the first time the differences in DNA methylation and RNA expression between Tibetan and Han T2DM patients, and synthesized the relationship between them, which provides a basis for further exploration of T2DM development mechanisms and identification of therapeutic targets.

Tibetans live in a high altitude, low oxygen, low temperature environment. Previous studies have shown that in a healthy population of Han Chinese and Tibetans living at the same altitude, the hemoglobin concentration of highland Han is higher than that of Tibetans (32). However, Han Chinese living at lower altitudes have lower hemoglobin concentrations (33), which is consistent with our results. The diet of Tibetans consists mainly of coarse grains, meat, yak butter and other high-fat, high-calorie, high-protein foods, thus have a higher BMI. However, no differences were shown in other lipid indicators in our cross-sectional cohort, except for higher LDL level in the Tibetan T2DM group, which indicates that Tibetans may have higher metabolism.

The Han and Tibetan populations also showed slight differences in liver function, with Tibetans having higher ALT and GGT levels, but both in the normal range. Aminotransferases are considered indicators of hepatocyte health, and GGT also reflects biliary tract function. Elevated ALT is associated with age, obesity, elevated triglyceride levels, and low HDL cholesterol levels, but not with glycemic control (34). However, independent of common risk factors, ALT (35, 36) and GGT (37) are linked to an increase in the risk of T2DM. Although the eGFR level of Tibetan T2DM patients was higher and the BUN was lower than that of Han Chinese, both were at normal levels.

In the exploratory cohort, we further investigated the differences in DNA methylation and transcriptome between Han and Tibetan populations to interpret the differences in the development of T2DM between the two groups through a genetic perspective. The CpG island is a region of the DNA sequence rich in CpG sites, usually located in the promoters with an unmethylated state. When CpG islands are methylated, transcription factors become impaired in binding to promoters or bind to transcriptional repressors, altering the structure of chromatin. As a result, gene expression is altered without the changes of DNA sequence, affecting biological processes and leading to diseases (38, 39).

In our study, a total of 1613 genes with DMRs were found between Han Chinese and Tibetan T2DM patients. After GO and KEGG functional enrichment analysis, we identified signaling pathways that affect metabolism and other pathways that may play a key role in the development of T2DM, such as insulin secretion. Among them, cAMP signaling pathway, Wnt signaling pathway, and Hippo signaling pathway were more significant and relevant. cAMP is an intracellular mediator of insulin and adrenal glycogen catabolism in the liver (40). In mammals, cAMP activates cAMP-dependent protein kinase (PKA), which phosphorylates downstream protein targets and then regulates the function of ion channels, transcription factors and enzymes. Meanwhile, the cAMP signaling pathway regulates glucose homeostasis due to insulin secretion, glucose utilization, and glycogen synthesis and catabolism (41). The Wnt signaling plays an important role as an evolutionary pathway in regulating cellular homeostasis and energy homeostasis from the hypothalamus to the metabolic organs. The classical Wnt as well as non-classical Wnt pathways inhibit metabolism and lead to increased adipose tissue, resulting in metabolic stress and metabolic inflammation and obesity (42). The Hippo signaling pathway plays a role in pancreatic, hepatic, adipose and cardiac cells as well as in systemic metabolism, regulating glucolipid metabolism. Activation of the Hippo signaling pathway in hyperglycemic states induces proliferation and differentiation of pancreatic β-cells, increasing glucose uptake and utilization, thereby reducing insulin resistance, and improving insulin secretion (43).

In general, gene expression follows an opposite trend to the level of methylation in the promoter region. In the present study, we identified 947 differentially expressed genes, of which 112 overlapping genes had differential methylation levels, and a total of 14 genes with differentially methylated regions in the promoter region. Among the differentially expressed genes found to be differentially methylated in promoter regions, *APOB* encodes apolipoprotein B and is associated with LDL, celiac and LDL structural integrity, in lipid digestion, mobilization as well as transport (44). A study on the amount of non-insulin-dependent diabetic patients showed that *APOB* polymorphisms were effective in improving blood glucose and lipid levels of T2DM patients (45). *PAX8-AS1* is a non-coding RNA that is involved in the pathology of the disease despite its inability to encode protein synthesis. In a study examining non-coding RNA in leukocytes from patients with gestational diabetes mellitus (GDM), *PAX8-AS1* expression levels were significantly lower in GDM patients compared to healthy pregnant women and could be used as a diagnostic biomarker for GDM (46). The rest of key genes need to be further studied in the future.

Similar to previously described, KEGG analysis was mainly enriched in metabolic pathways that are related to IR or diabetes, including metabolism of xenobiotics by cytochrome P450 (47), steroid hormone biosynthesis (48), retinol metabolism (49), ascorbate and aldarate metabolism (50), pentose and glucuronate interconversions (51), starch and sucrose metabolism. Several canonical pathways outstood among the statistics, including PI3K-Akt pathway, MAPK pathways and Rap1 signaling pathway. Insulin secretion activates PI3K-Akt signaling pathway throughout the body to increase glucose utilization, reduce glucose metabolism in the liver and muscle, and regulate the balance of lipid and glucose metabolism. However, impairment of this pathway leads to insulin resistance, which in turn worsens this pathway, leading to T2DM (52). In addition, insulin can also activate MAPK pathways but inappropriate MAPK signaling contributes to the development of metabolic syndrome and T2DM (53). An *in vitro* study showed that activated Rap1 is a key regulator of β-cell function, as evidenced by the promotion of glucose-stimulated insulin production, islet cell hypertrophy, and islet cell proliferation by activated Rap1A (54).

The overlapping genes primarily are associated with metabolism and insulin-related pathways, suggesting that the environment and lifestyle, such as diet, may play a role in altering DNA methylations levels, therefore affecting metabolism and insulin secretion and utilization in T2DM patients.

## Conclusion

As the prevalence of T2DM varies in different ethnic groups in China, our study revealed the diverse clinical features of Tibetan and Han T2DM patients. The epigenetic and transcriptional patterns have provided a perspective on the mechanisms of T2DM in different ethnic groups, and the key genes are worthy to be further studied to reveal the importance of DNA methylation for the development of T2DM.

## Data availability statement

The datasets presented in this study can be found in online repositories. The name of the repository/repositories and accession number(s) can be found below: https://www.ncbi.nlm.nih.gov/, accession number: PRJNA911064.

## Ethics statement

The studies involving human participants were reviewed and approved by Hospital of Chengdu University of Traditional Chinese Medicine. The patients/participants provided their written informed consent to participate in this study.

## Author contributions

JL and XW conceived of the presented idea, conducted the study of cross-sectional cohort. XW carried out the exploratory study of epigenome and transcriptome. QC and QW supervised the project. All the authors contributed to the final version of the manuscript. 

## Supplementary material

Supplementary Figure 1 The proportion of different types of methylated cytosines in 12 samples In the pie chart, red, green and blue indicate mCG, mCHH and mCHG, respectively. H: Han, Z: Tibetan

Supplementary Figure 2 Distribution of mC levels in mCG, mCH and mCHH. The figure showed the distribution of mC levels in 12 samples. The X axis showed the methylation level, and the Y axis showed the percentage of mC. Red, blue and green lines represent CG, CHG and CHH, respectively. H: Han, Z: Tibetan

Supplementary Figure 3 Distribution of DNA methylation levels in each gene elements. Red, blue and green lines represent CG, CHG and CHH, respectively. H: Han, Z: Tibetan
