# Supplementary figures and images for: The transcriptomic and epigenetic alterations in type 2 diabetes mellitus patients of Chinese Tibetan and Han populations
Source: Front Endocrinol (Lausanne). 2023 Feb 16;14:1122047. doi: 10.3389/fendo.2023.1122047 (PMC9987421; doi:10.3389/fendo.2023.1122047)

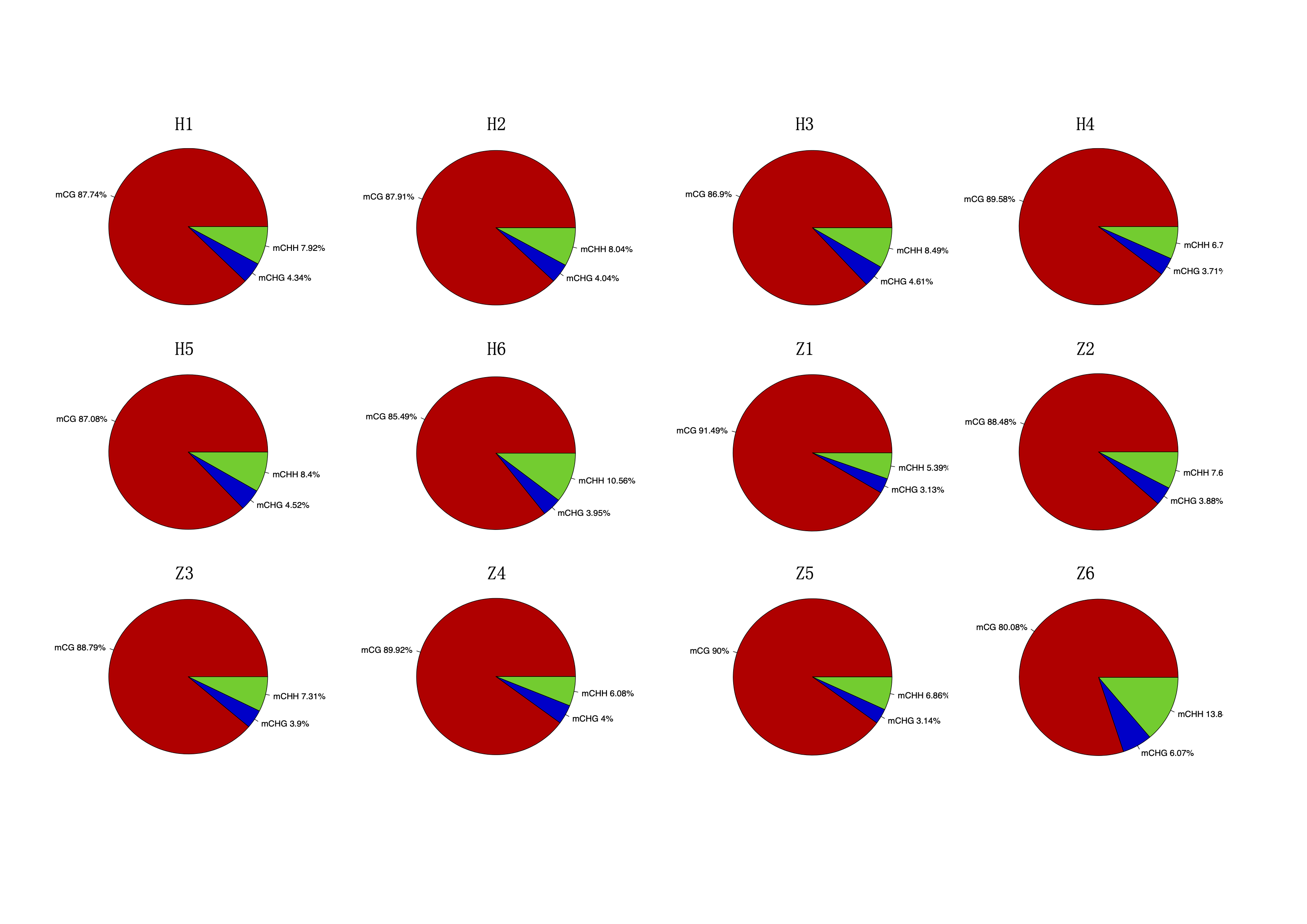

Supplement: Supplementary Figure 1 — The proportion of different types of methylated cytosines in 12 samples In the pie chart, red, green and blue indicate mCG, mCHH and mCHG, respectively. H: Han, Z: Tibetan [file Image_1.tiff]

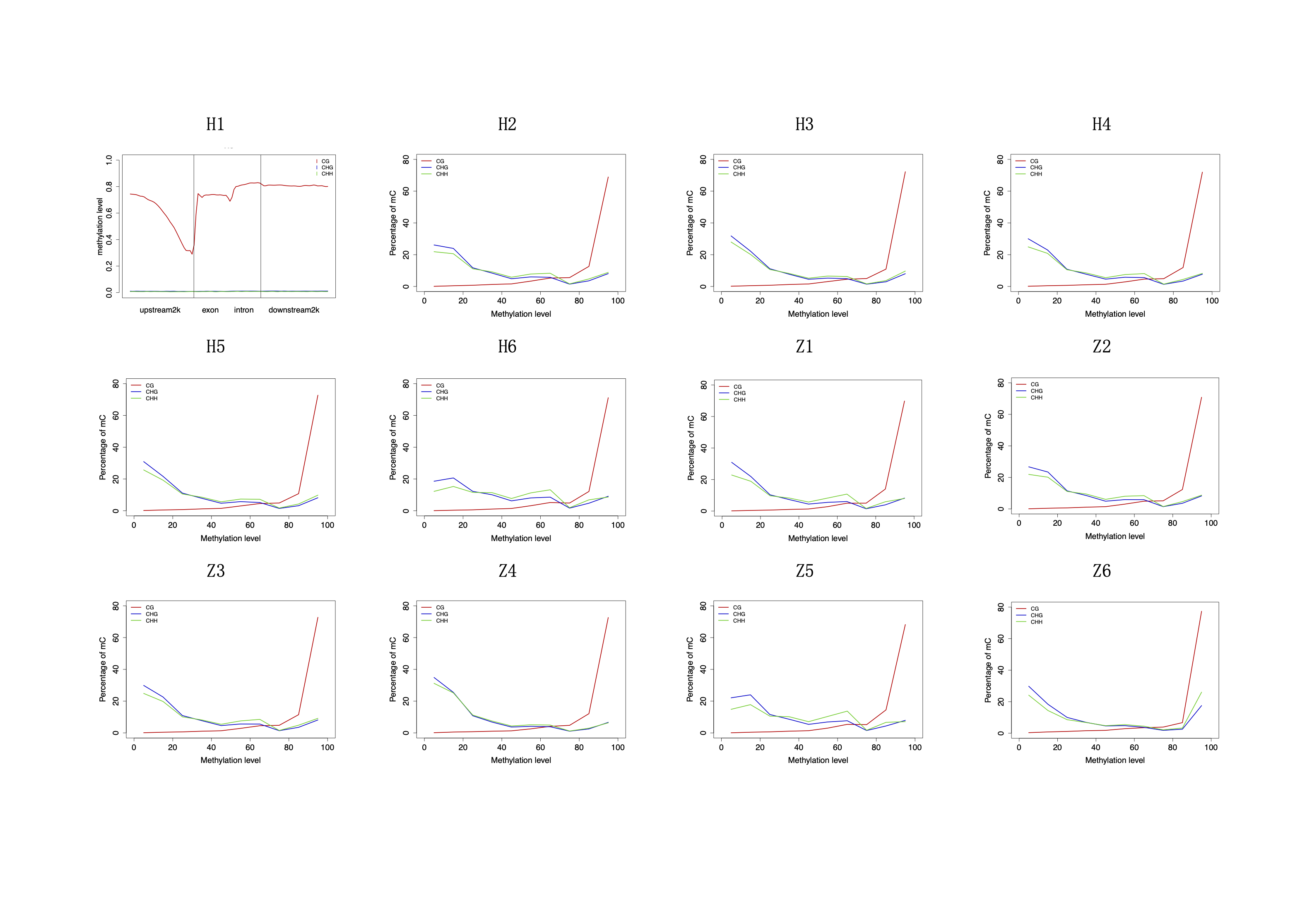

Supplement: Supplementary Figure 2 — Distribution of mC levels in mCG, mCH and mCHH. The figure showed the distribution of mC levels in 12 samples. The X axis showed the methylation level, and the Y axis showed the percentage of mC. Red, blue and green lines represent CG, CHG and CHH, respectively. H: Han, Z: Tibetan [file Image_2.tiff]

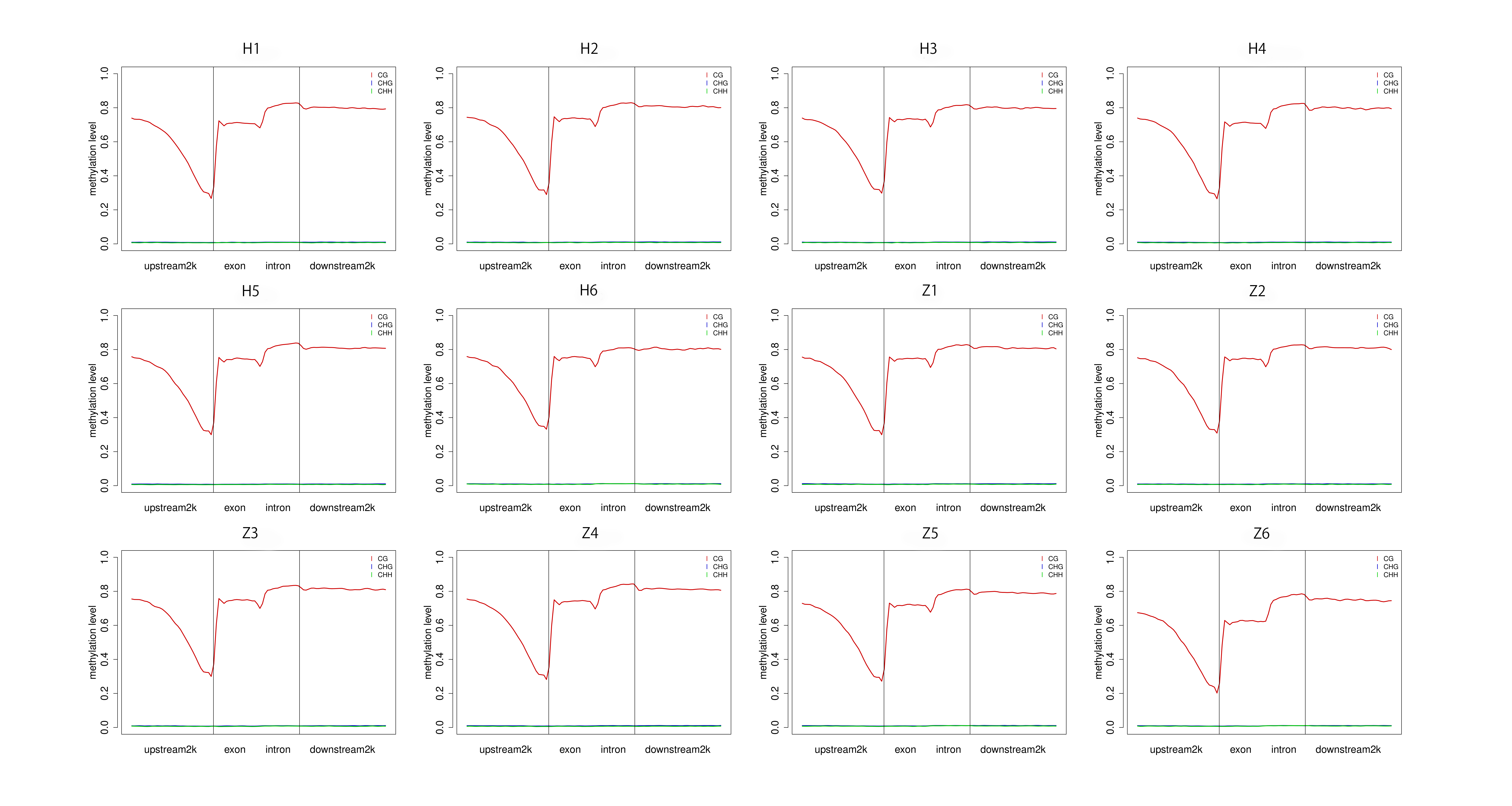

Supplement: Supplementary Figure 3 — Distribution of DNA methylation levels in each gene elements. Red, blue and green lines represent CG, CHG and CHH, respectively. H: Han, Z: Tibetan [file Image_3.tif]
