# Supplementary material for: The transcriptomic and epigenetic alterations in type 2 diabetes mellitus patients of Chinese Tibetan and Han populations
Source: Front Endocrinol (Lausanne). 2023 Feb 16;14:1122047. doi: 10.3389/fendo.2023.1122047 (PMC9987421; doi:10.3389/fendo.2023.1122047)
Supplement: Supplementary file 4 [file Table_1.docx]

**Supplementary Table S1. Demographical and biochemical characteristics between Tibetan and Han male T2DM patients in exploratory cohort**

|  | Tibetan（n = 6） | Han（n = 6） | P value |
| --- | --- | --- | --- |
| Age (years) | 43 (39-47.75) | 44 (40.5-53.25) | 0.246 |
| Duration of T2D (years) | 2.85 (1.65-5.08) | 3.7 (1.83-6.55) | 0.345 |
| BMI (kg/m^2^) | 23.13 (21.25-28.38) | 24.77 (23.86-27.68) | 0.116 |
| Hb (g/L) | 158.5 (142-163.75) | 153 (136.5-157.5) | 0.173 |
| HbA1c (%) | 9.9 (9.35-11.05) | 9 (8.25-9.5) | 0.028* |
| FBG (mmol/L) | 8.48 (6.98-10.06) | 9.99 (7.33-12.29) | 0.028* |
| 1-hr PBG (mmol/L) | 13.53 (12.31-16.2) | 16.37 (13.96-19) | 0.075 |
| 2-hr PBG (mmol/L) | 17.14 (15.14-18.75) | 18.25 (15.52-21.86) | 0.249 |
| 3-hr PBG (mmol/L) | 15.65 (13.3-17.88) | 17.79 (13.11-21.52) | 0.249 |
| 0-hr Insulin (mIU/L) | 11.81 (2.61-22.94) | 12.85 (6.51-45.53) | 0.917 |
| 1-hr Insulin (mIU/L) | 26.88 (10.16-46.6) | 36.11 (22.91-78.79) | 0.463 |
| 2-hr Insulin (mIU/L) | 34.53 (12.03-77.72) | 41.74 (24.28-91.74) | 0.991 |
| 3-hr Insulin (mIU/L) | 30.99 (12.51-85.32) | 31.21 (21.84-100.94) | 0.917 |
| 0-hr C-peptide (nmol/L) | 0.65 (0.42-1.26) | 0.79 (0.53-1.1) | 0.917 |
| 2-hr C-peptide (nmol/L) | 1.32 (1.09-2.44) | 1.71 (1.39-2.03) | 0.753 |
| TC (mmol/L) | 3.79 (3.48-4.19) | 4.64 (3.82-5.73) | 0.173 |
| TG (mmol/L) | 1.28 (0.99-2.18) | 1.17 (1.03-4.03) | 0.753 |
| HDL (mmol/L) | 0.94 (0.77-1.08) | 1.17 (1-1.45) | 0.046* |
| LDL (mmol/L) | 2.58 (2.13-2.83) | 2.46 (1.96-2.8) | 0.833 |
| ALT (IU/L) | 33.5 (20-57) | 24 (17.5-47.5) | 0.917 |
| AST (IU/L) | 22 (19.75-23.25) | 18.5 (15.75-27.25) | 0.344 |
| ALP (IU/L) | 72.5 (47.75-79.75) | 83.5 (71.25-95.75) | 0.345 |
| GGT (IU/L) | 44 (20.5-89.75) | 19 (10.75-56.75) | 0.173 |
| TBA (μmol/L) | 7.25 (3.35-8.03) | 8.4 (4.13-11.7) | 0.173 |
| DBIL (μmol/L) | 11.7 (8.45-16.2) | 13.15 (8.98-24.83) | 0.463 |
| BCr (μmol/L) | 58.95 (49-64.48) | 66.3 (50.48-81.6) | 0.249 |
| BUN (mmol/L) | 5.11 (3.44-5.98) | 5.2 (4.56-7.33) | 0.345 |
| BUA (μmol/L) | 289.5 (232.25-344.5) | 346.5 (267-396.75) | 0.345 |
| eGFR (mL/min) | 143.7 (109.6-179.3) | 97 (66.56-102.43) | 0.046* |

HbA1c: hemoglobin A1c, FBG: fasting blood glucose, PBG: post-prandial blood glucose, TC: total cholesterol, TG: triglycerides, HDL: high-density lipoprotein, LDL: low-density lipoprotein, ALT: alanine aminotransferase, AST: aspartate transaminase, ALP: alkaline phosphatase, GGT: γ-glutamyl transpeptidase, TBA: total bile acid, DBIL: direct bilirubin, BCr: blood creatinine, BUN: blood urea nitrogen, BUA: blood uric acid, eGFR: estimated glomerular filtration rate. Data were shown as median (25% - 75%).
