# Supplementary material for: The transcriptomic and epigenetic alterations in type 2 diabetes mellitus patients of Chinese Tibetan and Han populations
Source: Front Endocrinol (Lausanne). 2023 Feb 16;14:1122047. doi: 10.3389/fendo.2023.1122047 (PMC9987421; doi:10.3389/fendo.2023.1122047)
Supplement: Supplementary file 5 [file Table_2.docx]

**Supplementary table S2. Summary of RNA sequencing quality and read alignment to the reference genome.**

| Sample | Raw bases | Clean bases | Clean rate | Q30 | Effective Reads | Total Mapped | Uniquely Mapped | Reads Mapped in Proper Pairs |
| --- | --- | --- | --- | --- | --- | --- | --- | --- |
| H1 | 7.99G | 7.56G | 94.68% | 90.76% | 51,870,274 | 92.12% | 88.51% | 89.14% |
| H2 | 8.47G | 8.96G | 95.25% | 90.67% | 54,951,084 | 90.38% | 86.98% | 87.25% |
| H3 | 9.72G | 9.24G | 95.05% | 90.54% | 63,292,130 | 93.08% | 90.05% | 90.21% |
| H4 | 9.11G | 8.64G | 94.87% | 90.63% | 59,368,960 | 92.19% | 89.05% | 89.73% |
| H5 | 10.92G | 10.42G | 95.43% | 90.52% | 70,889,694 | 93.18% | 89.81% | 89.98% |
| H6 | 7.67G | 7.24G | 94.39% | 90.00% | 49,808,842 | 90.74% | 87.54% | 88.03% |
| Z1 | 12.15G | 11.51G | 94.79% | 90.80% | 79,189,106 | 91.28% | 87.64% | 88.58% |
| Z2 | 6.39G | 6.02G | 94.26% | 90.34% | 41,391,154 | 90.13% | 87.15% | 87.41% |
| Z3 | 8.75G | 8.32 | 95.15% | 90.43% | 56,903,340 | 93.22% | 89.50% | 90.49% |
| Z4 | 8.63G | 8.16G | 94.58% | 91.01% | 56,176,772 | 90.58% | 87.76% | 87.90% |
| Z5 | 6.54G | 6.19G | 94.70% | 90.37% | 45,664,690 | 90.29% | 87.14% | 88.23% |
| Z6 | 6.56G | 6.23G | 94.99% | 90.82% | 52,261,856 | 75.26% | 72.21% | 71.87% |
| Average | 8.575G | 8.32G | 94.85% | 90.57% | 56,813,992 | 90.2% | 86.95% | 87.4% |

H: Han, Z: Tibetan
