# Supplementary material for: The transcriptomic and epigenetic alterations in type 2 diabetes mellitus patients of Chinese Tibetan and Han populations
Source: Front Endocrinol (Lausanne). 2023 Feb 16;14:1122047. doi: 10.3389/fendo.2023.1122047 (PMC9987421; doi:10.3389/fendo.2023.1122047)
Supplement: Supplementary file 6 [file Table_3.docx]

**Supplementary Table S3. Association between promoter-related overlapping genes and significantly different biochemical parameters.**

|  | HbA1c | FBG | 3-hr Insulin | HDL | eGFR |
| --- | --- | --- | --- | --- | --- |
| AJAP1 | 0.394 | 0.222 | -0.589* | -0.377 | 0.460 |
| APOB | 0.055 | -0.631* | -0.187 | -0.428 | 0.320 |
| COL1A1 | 0.154 | 0.034 | -0.192 | -0.235 | 0.096 |
| FOXA1 | 0.175 | -0.340 | -0.477 | -0.530 | 0.794** |
| MIXL1 | -0.374 | 0.062 | 0.830*** | 0.286 | -0.389 |
| MYCN | 0.309 | -0.200 | -0.408 | -0.213 | 0.339 |
| OXCT2 | -0.018 | 0.104 | 0.663** | -0.098 | -0.363 |
| RHOD | 0.697* | -0.208 | -0.517 | -0.530 | 0.524 |
| LAMA5-AS1 | -0.524 | -0.123 | 0.910*** | 0.086 | -0.256 |
| LOC100134868 | -0.727** | -0.028 | 0.621* | 0.284 | -0.291 |
| LOC102723672 | -0.661* | 0.064 | 0.758** | 0.115 | -0.397 |
| LOC102723828 | -0.026 | -0.022 | 0.233 | 0.394 | -0.401 |
| PAX8-AS1 | -0.264 | 0.468 | 0.164 | 0.615* | -0.370 |
| UMODL1-AS1 | 0.547 | -0.046 | -0.452 | -0.429 | 0.662* |

HbA1c: hemoglobin A1c, FBG: fasting blood glucose, HDL: high-density lipoprotein, eGFR: estimated glomerular filtration rate. * P < 0.05, ** P < 0.01, *** P < 0.001.
